# Analytical Validation and Performance Evaluation of Amplicon-Based Next-Generation Sequencing Assays for Detecting *ERBB2* and Other Gene Amplifications in Solid Tumors

**DOI:** 10.3390/cancers16233927

**Published:** 2024-11-23

**Authors:** Ekaterina Olkhov-Mitsel, Danny Chan, Kenneth J. Craddock, August Lin, Grace Luk, Rashmi S. Goswami, Hong Wang, Anna Plotkin, Sharon Nofech-Mozes, David M. Hwang, Weei-Yuarn Huang

**Affiliations:** 1 Precision Diagnostics and Therapeutics Program, Division of Anatomic Pathology, Department of Laboratory Medicine and Molecular Diagnostics, Sunnybrook Health Sciences Centre, Toronto, ON M4N 3M5, Canada; 2 Department of Laboratory Medicine and Pathobiology, Temerty Faculty of Medicine, University of Toronto, Toronto, ON M5S 1A8, Canada; 3 Biological Sciences Platform, Sunnybrook Research Institute, Sunnybrook Health Sciences Centre, Toronto, ON M4N 3M5, Canada; 4 Department of Pathology and Laboratory Medicine, University of Western Ontario, London, ON N6A 3K7, Canada; 5 Genetics Program, North York General Hospital, Toronto, ON M2K 1E1, Canada

**Keywords:** amplicon based next-generation sequencing, amplicon-based panels, copy number variation, gene amplification, ERBB2 amplification, fluorescence in situ hybridization, solid tumors, molecular pathology

## Abstract

Background: Targeted next-generation sequencing (NGS) panels are increasingly being utilized to identify actionable gene amplifications (copy number > 4) among solid tumors. Methods: This study validated the analytical performance of two amplicon-based NGS assays, the Oncomine Comprehensive Panel (OCAv3) and the Oncomine Focus Assay (OFA), for detecting gene amplification in formalin-fixed paraffin-embedded (FFPE) tumors of varying cellularity. OCAv3 was assessed for amplification detection in 756 FFPE samples comprising various tumor types. Results: We demonstrated that with standardized quality control metrics, including median absolute pairwise difference score, these assays can achieve a near-perfect positive predictive value, although their sensitivity for detecting amplifications significantly decreased in tumors with cellularity below 30%. Stratifying tumor cellularity into 10–30%, 31–60%, and 61–95% groups revealed significantly higher gene amplification detection rates in the 31–60% and 61–95% groups *versus* the 10–30% group (20.6% and 26.7% vs. 9.2%, *p* < 0.0001). When considering all detected gene amplifications, the average amplification calling per sample was nearly five-fold lower in the 10–30% group *versus* the 61–95% group (0.11 vs. 0.52; *p* < 0.0001). To further investigate the analytic performance of OCAv3 in detecting *ERBB2* amplification, we analyzed a cohort of 121 uterine carcinomas with confirmed *ERBB2* status by HER2 IHC or FISH, in which a threshold incorporating amplifications and tumor cellularity achieved 79% sensitivity and 100% specificity, potentially eliminating the need for FISH analysis in 34% of equivocal cases. In a separate validation cohort, similar analytical performance was observed, with the threshold demonstrating consistent sensitivity and specificity. Conclusions: This study highlights the strengths and limitations of amplicon-based NGS assays in detecting amplifications using real-world data.

## Simple Summary

One way cancer develops is through gene amplification, where copies of certain genes increase. Traditionally, this has been detected using a technique called fluorescence in situ hybridization (FISH). However, newer methods like amplicon-based next-generation sequencing (NGS) are increasingly being used to detect DNA mutations and can also identify changes in gene copy numbers. However, NGS has not yet been validated for routine clinical use for detecting gene amplification. This study evaluated NGS as a less resource-intensive alternative to traditional methods like FISH. The findings highlight the strengths and limitations of using amplicon-based NGS assays for detecting gene amplification in preserved pathology tissue samples. By modifying the criteria for measuring gene copy numbers and considering tumor characteristics, this study aimed to enhance the accuracy of amplicon-based NGS in detecting gene amplifications with a specific focus on *ERBB2* gene amplification. This could potentially decrease the reliance on FISH testing and help improve testing strategies in clinical settings, ultimately guiding treatment decisions.

## 1. Introduction

The use of next-generation sequencing (NGS) panel analysis of DNA from formalin-fixed paraffin-embedded (FFPE) tissue is gaining popularity for identifying actionable copy number gains, or more specifically, gene amplifications, defined as copy number > 4 [1,2,3,4,5,6,7]. Compared to standard methods such as in situ hybridization (ISH) and array CGH, targeted NGS assays offer advantages in simultaneously detecting single nucleotide variants (SNVs) and small insertions/deletions (indels) with low DNA input requirements [8,9,10,11]. Additionally, they are less labor-intensive in data interpretation and fully optimized for FFPE samples. Colorectal cancer (CRC) and uterine serous carcinoma are among the major cancer types where NGS is advocated as the initial testing approach for detecting *ERBB2* amplification to identify eligible patients for targeted therapies [3,12,13]; however, the limitations, sensitivity, and specificity of various NGS panels in detecting such amplifications have not been adequately addressed in these studies [12,13].

Algorithms and bioinformatic pipelines for determining copy number variation(s) (CNVs) have been developed and established for NGS protocols based on hybridization-capture and whole exome sequencing or targeted large NGS panels [14,15]. In contrast, less information is available for CNV analysis in NGS data from smaller, amplicon-based NGS panels that have been widely used for determining sequencing variants in FFPE samples with limited amounts of DNA [2].

In amplicon-based NGS panels, detecting CNVs, especially gene amplifications, relies on calculating relative amplicon coverage and B-allele frequencies [2]. Various factors, such as sequencing quality metrics including the number of amplicons per gene, mean depth of coverage and tumor purity within the sample, can impact the accuracy of amplification detection [1]. Thus, both the assay along with the data analysis, interpretation and reporting algorithms need to be fully validated prior to clinical implementation.

In accordance with the Association for Molecular Pathology (AMP) guidelines for NGS test validation, we conducted analytical validation of two amplicon-based NGS panels—the Oncomine Focus Assay (OFA) and Oncomine Comprehensive Assay v3 (OCAv3)—for implementation in routine clinical practice at our institution. The assays demonstrated an overall 90% sensitivity and 100% specificity for the detection of DNA sequencing variants (SNV and small indels). However, gene amplification detection was not assessed during our initial clinical validation phase. Consequently, gene amplifications were not reported when we began routine solid tumor NGS testing at our institution in early 2021. In this study, we took an additional step to evaluate and validate the analytical performance of both NGS panels in detecting gene amplifications, with a specific focus on *ERBB2* amplification across various cancer types due to its clinical relevance in the context of targeted therapies.

## 2. Materials and Methods

### 2.1. Tumor Specimens, Commercial Reference Materials and DNA Preparation

**Figure 1:**
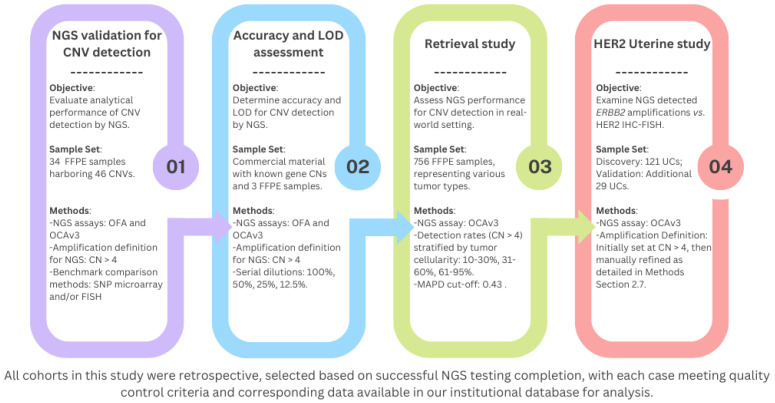
Study design and methodology flowchart. CNV, copy number variations; FFPE, formalin-fixed paraffin-embedded; FISH, fluorescence in situ hybridization; IHC, immunohistochemistry; LOD, limit of detection; MAPD, median absolute pairwise difference; NGS, next-generation sequencing; OCAv3, Oncomine Comprehensive Assay v3; OFA, Oncomine Focus Assay; SNP, single nucleotide polymorphism; UC, uterine carcinoma.

This retrospective study, approved by our institutional research ethics board, involved data retrieval from our institutional NGS assay validation database and archived tumor NGS results database. This study included four archival cohorts, comprising 34, 756, 121 and 29 cases, all retrospectively analyzed (Figure 1). These tumor samples underwent NGS testing between October 2021 and September 2024 as part of routine clinical practice at our institution for detecting DNA sequencing variants (SNVs and small indels). All cases met vendor-recommended quality control (QC) metrics, with a minimum tumor cellularity of 10% required for NGS assays. Eight uterine carcinoma cases overlapped between the 756- and 121-case cohorts.

Specimen processing followed standardized institutional protocols. The clinical laboratory timestamped the samples upon arrival from the operating room and the time formalin was added, typically within an hour of receipt. Cold ischemia times were recorded only for breast tissues, per provincial guidelines. Specimens were grossed and cassettes prepared the next day, following a 24–36 h fixation period.

Genomic DNA (gDNA) was extracted from archival FFPE tumor samples (10 slides, 5 micron each) using the Promega Maxwell RSC Instrument (Promega, Madison, WI, USA). The extracted gDNA was quantified via Qubit 3 Fluorometer using Qubit dsDNA (Thermo Fisher Scientific Inc., Waltham, MA, USA), and 10 ng of DNA was used for NGS.

For the benchmarking study, 34 tumor samples were included: 17 lung adenocarcinomas (NSCLC), nine endometrial adenocarcinomas, four CRCs, two melanomas, one bladder cancer and one breast cancer. Eighteen were tested via the Oncomine Focus Assay (OFA) using 520 chips and 16 were tested via the Oncomine Comprehensive Assay v3 (OCAv3) using 540 chips (Thermo Fisher Scientific Inc., Waltham, MA, USA). Two reference DNA samples (SeraSeq Breast CNV Mix-Cat # 0710-0416 and SeraSeq Lung & Brain CNV Mix Cat # 0710-0413) from SeraCare (Waterbeach, UK) with known amplifications in *ERBB2, FGFR3, MET*, *EGFR*, *MYCN,* and *MYC* were serially diluted by mixing with wild-type reference standard (Horizon Discovery–Cat # HD-709). Limit of detection experiments were performed in duplicate using the (diluted) DNA references and FFPE samples at 100%, 50%, 25%, and 12.5% to determine the lower limit of detection (LLOD) of both NGS assays for CNV. The tumor cellularity of the FFPE samples used was 10–90%.

### 2.2. Targeted NGS Assays and Bioinformatic Analysis

The targeted NGS assays were performed on the Ion S5XL next-generation sequencing system (Thermo Fisher Scientific Inc., Waltham, MA, USA). Amplicon library construction and sequencing template preparation were performed using Ion Chef with Ion 520 kit for OFA and Ion 540 kit for OCAv3. The OCAv3 panel detects SNVs, CNVs, gene fusions, and indels across 161 cancer driver genes. A full list of the genes included in this panel is available online (https://www.thermofisher.com/order/catalog/product/A35805). The OFA panel covers over 1,000 clinically relevant variants, including hotspots, SNVs, indels, CNVs, and gene fusions across 52 genes. A full list of the genes included in this panel is available online (https://www.thermofisher.com/order/catalog/product/A42008).

Seven tumor samples and one NTC (no template control) were included in each sequencing chip. The Ion Torrent platform-specific pipeline software, Torrent Suite version 5.18.0 (Thermo Fisher Scientific Inc., Waltham, MA, USA) was used to separate barcoded reads and to filter and remove polyclonal and low-quality reads. Bioinformatics analysis was performed using Ion Torrent platform specific bioinformatics software, Ion Reporter version 5.18, following standard operating procedures and the manufacturer’s recommended Oncomine extended filter chain (5.18) with a modification enhancing the detection of *MET* exon 14 skip. In this workflow, the software uses a default cellularity assumption of 100% for CNV calculations (Thermo Fisher Scientific Inc., Waltham, MA, USA). The overall quality of sequence data was assessed by number of mapped reads, % of on target reads, mean sequencing depth, mean sequencing length, uniformity, and median absolute pairwise difference (MAPD) score. For the results of each sequencing run to be considered acceptable, each of the above listed quality metrics was required to pass the vendor-recommended thresholds (Table 1).

### 2.3. Single Nucleotide Polymorphism Microarray Analysis

CNVs were assessed using the Infinium CytoSNP-850K BeadChip (Illumina, Inc., San Diego, CA, USA). In brief, 200 ng of gDNA from tumoral tissues was amplified and hybridized to the BeadChip. The stained BeadChip was then scanned on the NextSeq 550 (Illumina) and analyzed using NxClinical 6.0 software (Bionano Genomics, San Diego, CA, USA). CN amplification by SNP microarray was called based on established algorithm thresholds, CN >4, as determined by the NxClinical 6.0 software.

### 2.4. HER2 Immunohistochemistry

HER2 immunohistochemistry (IHC) staining was performed on whole-slide tissue sections using the Ventana 4B5 antibody (Roche), following established clinical standards. Slides were assessed by one of two expert gynecologic and breast pathologists and scored based on the 2020 ISGyP recommendations [16]. Endometrial tumors were categorized as HER2 3+ if they showed strong basolateral/lateral or circumferential membranous HER2 staining in >30% of tumor cells. A score of 2+ (equivocal) was assigned if strong staining was observed in 30% or fewer tumor cells, or showed weak to moderate membranous staining in at least 10% of tumor cells, followed by reflex *ERBB2* in situ hybridization (ISH) to evaluate gene amplification, as defined below (Section 2.5). A score of 1+ indicated faint or barely perceptible incomplete membrane staining in any proportion, or weak complete staining in <10% of tumor cells. Cases with no staining were scored as 0.

### 2.5. ERBB2 Fluorescence in Situ Hybridization

In the uterine carcinoma cohorts, tumor samples underwent dual-probe fluorescence in situ hybridization (FISH) as part of routine clinical practice at our institution to detect *ERBB2* amplification using one of two validated protocols in our clinical laboratory, depending on reagent availability, which was affected by the COVID-19 pandemic: (1) PathVysion *HER-2* DNA Probe kit (Vysis, Dover, IL, USA); (2) automated *HER2* IQFISH pharmDx (Dako Omnis) assay and the Duet-3/Setup Station/SOLO2 system (BioView, Rehovot, Israel). Both were performed in accordance with established clinical standards. Experienced gynecologic and breast pathologists assessed whole-section endometrial cancer samples for *ERBB2* amplification based on the 2020 ISGyP recommendations [16]. *ERBB2* amplification was defined, in accordance with established guidelines by the College of American Pathologists (CAP), as *ERBB2*/*CEP17* ratio ≥ 2.0 or an average *ERBB2* copy number ≥ 6 per nucleus, with the entire tumor area evaluated and enumeration conducted in a minimum of 20 contiguous cells forming a tight cluster within the most intensely stained tumor areas identified by DAPI-filtered fluorescent microscopy. Pathologists specifically examined areas exhibiting the highest HER2 IHC intensity and formally counted *ERBB2* gene signals where signal variations were observed. Enumeration and calculation of the FISH ratio were performed using TRITC filter or Spectrum Orange (SO) filter (red) and FITC filter (green) for *ERBB2* and *CEP17* signals, respectively.

### 2.6. Tumor NGS Database Retrieval and Statistical Analysis

NGS data obtained from 756 tumor samples tested with the OCAv3 assay between October 2021 and August 2022 in our institution were retrieved and reviewed. Tumor purity was retrieved from pathology reports. As part of routine clinical workflow, all tumors were assessed by four pathologists with subspecialty training in either molecular pathology, gastrointestinal pathology and/or pulmonary pathology. Tumor samples included 242 CRCs, 142 lung cancers, 135 endometrial cancers, 153 melanomas, 63 prostate cancers, 13 breast cancers, two biliary cancers, one bladder cancer, four glioma and one thyroid cancer. A threshold of  >4 copies was used to define CN amplification.

### 2.7. Definitions of Copy Number Amplification

CN amplification by targeted amplicon-based NGS assays was defined as CN > 4, as determined by the Ion Torrent platform-specific bioinformatics software, Ion Reporter version 5.18. For a subsequent sub-analysis of the analytical performance of OCAv3 NGS in detecting *ERBB2* amplification in uterine carcinoma (results Section 3.5), the CN threshold and tumor cellularity cutoffs were manually refined to improve sensitivity. Specifically, samples with a CN of 2.6 or less were classified as negative, regardless of tumor cellularity. For CN values between 2.6 and 3.7, samples were considered positive if tumor cellularity was 75% or less and negative if tumor cellularity exceeded 75%. Samples with a CN of 3.7 or greater were classified as positive, irrespective of tumor cellularity.

Amplicon-based NGS assays were benchmarked against the gold standard methods for gene amplification detection currently used in laboratories, including SNP microarray and FISH. CN amplification by SNP microarray was called based on established algorithm thresholds, CN > 4, as determined by the NxClinical 6.0 software.

*ERBB2* amplification by FISH was defined as *ERBB2*/*CEP17* ratio ≥ 2.0 or an average *ERBB2* copy number ≥ 6 per nucleus, with evaluation of the entire tumor area and enumeration in a minimum of 20 contiguous cells forming tight clusters within the most intensely stained tumor regions, in accordance with established CAP guidelines.

### 2.8. Statistical Analysis

Continuous and categorical variables were compared using *t*-test and Fisher’s exact test, respectively. A *p*-value < 0.05 was considered to be statistically significant. OCAv3 assay sensitivity (true-positive results divided by true-positive results plus false-negative results), specificity (true-negative results divided by true-negative results plus false-positive results), positive predictive value (PPV; true-positive results divided by total positive results), and negative predictive value (NPV, true-negative results divided by total negative results) compared to IHC/FISH were reported.

## 3. Results

### 3.1. Comparing Amplification Calling by NGS to Single Nucleotide Polymorphism Microarray and ERBB2 FISH

To evaluate the analytical performance of gene amplification detection by targeted amplicon-based NGS assays, a first set of 34 FFPE retrospective samples that harbored a total of 46 amplifications were selected for the benchmarking study. Table 1 presents the sequencing QC metrics for these samples, including mean depth of coverage, uniformity percentage, mean read length and MAPD scores.

Of the 34 samples tested by NGS, 28 had sufficient DNA for SNP microarray testing for the benchmarking study. Among these 28 samples, two were also tested using FISH. The remaining six samples did not have sufficient DNA for SNP microarray testing and were therefore assessed only by FISH. Of the 41 gene amplifications detected by NGS (defines as CN > 4), 38 were also detected by SNP microarray (Table 2). Differences in copy number output between NGS and SNP microarray were attributed to variations in tumor DNA purity, methodology (amplicon read counts vs. fluorescence probes), resolution (gene-level vs. broader regions), and bioinformatics algorithms. Despite these differences, the final qualitative calls—positive or negative for amplification—were largely consistent between the two assays.

**Table 2:** Amplification calls from next-generation sequencing, compared to SNP microarrays for all analyzed genes.

Sample	Tumor Cellularity	NGS Panel	Gene	NGS Copy Number	SNP Microarray Copy Number
1	65	OFA	*CDK4*	5.5	5.0
2	35	OFA	*FGFR1*	5.6	5.0
			*KRAS*	9.3	18.0
3	70	OFA	*MET*	5.7	10.0
			*EGFR*	**3.8**	**4.0**
4	80	OFA	*EGFR*	11.4	5.0
5	90	OFA	*EGFR*	15.2	7.0
			*CCND1*	5.1	5.0
6	30	OFA	*EGFR*	35.7	15.0
7	50	OFA	*CDK4*	7.8	6.0
			*MYC*	**3.1**	**4.0**
8	70	OFA	*MYC*	13.6	7.5
9	70	OFA	*MET*	11.3	4.0
10	80	OFA	*MET*	5.2	7.7
11	90	OFA	*MET*	7.6	7.8
12	30	OFA	*ERBB2*	26.4	4.4
13	20	OFA	*ERBB2*	11.9	5.6
14	80	OFA	*EGFR*	8.5	5.7
15	50	OFA	*EGFR*	8.7	9.2
16	50	OFA	*EGFR*	46.6	30.7
17	80	OCAv3	*FGFR1*	6.0	7.0
			*CCND1*	5.5	4.0
			*NBN*	4.9	7.0
			*MYC*	4.2	7.0
18	95	OCAv3	*TERT*	7.6	4.0
			*HIST1H3B*	8.3	7.0
			*BRAF*	4.9	10.0
			*EZH2*	6.4	10.0
19	90	OCAv3	*TERT*	5.4	5.0
20	90	OCAv3	*CCNE1*	30.6	5.0
21	85	OCAv3	*CCNE1*	7.3	4.0
22	80	OCAv3	*CCNE1*	**5.9**	**3.7**
23	80	OCAv3	*CCNE1*	13.1	4.0
24	65	OCAv3	*NBN*	4.7	7.2
			*CDKN1B*	5.0	5.8
			*MYC*	4.8	7.2
			*KRAS*	4.3	5.8
25	75	OCAv3	*ERBB2*	36.2	5.0
			*NBN*	**3.0**	**4.0**
			*MYC*	**2.9**	**4.0**
26	75	OCAv3	*ERBB2*	20.7	5.0
			*MYC*	**3.6**	**5.0**
27	80	OFA	*ERBB2*	**6.6**	**3.0** *
			*BRCA 1*	6.8	6.7
28	90	OCAv3	*ERBB2*	**4.8**	**2.0** *
			*CCNE1*	19.8	5.0

NGS, next-generation sequencing; SNP, single nucleotide polymorphism; OCAv3, Oncomine Comprehensive Panel; OFA, Oncomine Focus Assay. Genes not covered by OFA or OCAv3 but detected by SNP were not listed. * FISH *ERBB2*/CEP17 ratio > 2.

**Table 3:** *ERBB2* amplification calls from next-generation sequencing, compared to SNP microarrays and fluorescence in situ hybridization.

Sample #	NGS Panel	NGS Copy Number	SNP Copy Number	FISH *ERBB2*/*CEP17* Ratio
27	OFA	6.6	3	≥2
28	OCAv3	4.8	2	≥2
29	OFA	26.6	N/A	≥2
30	OCAv3	26.6	N/A	≥2
31	OCAv3	9.4	N/A	≥2
32 *	OCAv3	17.6	N/A	≥2
33 *	OCAv3	8.1	N/A	≥2
34	OCAv3	86.2	N/A	≥2

* Sample # 32 and 33 had MAPD scores close to the upper limit set by the vendor. Both were amplified by FISH; see discussion. FISH, fluorescence in situ hybridization; NGS, next-generation sequencing; SNP, single nucleotide polymorphism; OCAv3, Oncomine Comprehensive Panel; OFA, Oncomine Focus Assay.

Of note, four samples harboring *ERBB2* amplification (CN > 4) detected by NGS were confirmed by SNP array (samples 12, 13, 25, 26). Two of the three amplifications (two *ERBB2* and one *CCNE1*) not detected by SNP array were *ERBB2* amplifications (samples 27 and 28, CN < 4). These two samples, along with six other samples (samples 29 to 34), were subsequently confirmed to be positive for *ERBB2* amplification by FISH (*ERBB2*/*CEP17* ratio ≥ 2.0) (Table 3). Thus, comparing to SNP microarray and FISH results, NGS achieved a perfect positive predictive value (100%) for detecting *ERBB2* amplification. We were unable to determine if the *CCNE1* amplification detected in sample 22 (Table 2) by NGS was a false positive call by NGS or a false negative result by SNP microarray. This uncertainty is compounded by the fact that both methods provide CN estimates that are influenced by tumor cellularity. There were five amplifications from four samples identified by the SNP microarray, but not by NGS (Table 2, samples 3, 7, 25 and 26). These five amplifications (three *MYC*, one *EGFR,* and one *NBN*) missed by NGS but detected by SNP microarray were considered false negatives by NGS, generally representing low-level amplifications (four to five copies). Therefore, overall sensitivity of NGS was 88.9% and PPV 97.6%.

### 3.2. Evaluation of Amplification Calling by NGS Using Commercial Reference Materials and FFPE Samples

To determine the accuracy and LOD of amplifications, we tested reference materials with known copy numbers across six cancer driver genes (*EGFR*, *ERBB2*, *FGFR3 MET*, *MYC* and *MYCN*). The results indicate that both OFA and OCAv3 assays can accurately determine copy numbers (Table 4). When performing serial dilutions of samples with a gene copy number of 14 (assuming 100% tumor cellularity in undiluted DNA material), both OCAv3 and OFA assays detected amplifications (4 copies and above) when tumor cellularity was greater than 25.0% (Table 4).

We further validated this by performing the same dilutions (100%, 50%, 25%) in three FFPE samples with a mean *ERBB2* CN of 16 (range 9–24) and a mean MAPD score of 0.25 ± 0.091. Similar to the reference materials, a consistent decrease in CN was observed with each dilution, demonstrating the robustness of the assay. These results confirmed that the LOD is applicable to both reference materials and FFPE samples.

### 3.3. Rate of Amplification Detection Across Different Tumor Types Stratified by Tumor Cellularity

We next explored the real-world performance of the OCAv3 assay, which enables the detection of CNVs in a panel of 43 genes, including *AKT1, AKT2, AKT3, ALK, AXL, AR, BRAF, CCND1, CCND2, CCND3, CCNE1, CDK2, CDK4, CDK6, EGFR, ERBB2, ESR1, FGF19, FGF3, FGFR1, FGFR2, FGFR3, FGFR4, FLT3, IGF1R, KIT, KRAS, MDM2, MDM4, MET, MYC, MYCL, MYCN, NTRK1, NTRK2, NTRK3, PDGFRA, PDGFRB, PIK3CB, PIK3CA, PPARG, RICTOR* and *TERT*. The performance of OCAv3 in detecting gene amplifications (CN > 4) was evaluated across various tumor types, with the detection rates summarized in Table 5. The cohort consisted of 756 cases; however, only 748 cases are listed in Table 5, as the remaining eight cases represent rare occurrences of specific cancers (biliary, bladder, glioma, and thyroid).

Overall, CNV detection ranged from 0 up to 6 per sample. At least one amplification (CN > 4) was detected in 20.0% of tumors, with frequent occurrences observed in breast (38.5%) and endometrial cancers (31.1%). Melanoma exhibited the lowest detection rate at 11.8%. In CRC, *BRCA2* and *FLT3* amplifications were relatively common at 6.6% and 4.5%, respectively. *EGFR* amplification was predominantly found in lung cancer (3.5%). About 10% of endometrial cancers harbored *ERBB2* amplification, contrasting with 2.0% in CRC and lung cancer (*p* = 0.0002).

Given that the sensitivity of NGS in detecting amplification (CN > 4) depends on tumor cellularity, we further assessed the impact of varying tumor cellularity on the rate of amplification detection (Table 6).

When stratifying tumor cellularity into 10–30%, 31–60% and 61–95% groups, the rate of detection of at least one amplification (CN > 4) in each sample was significantly higher in 31–60% and 61–95% groups compared to the 10–30% group (20.6% and 26.7% vs. 9.2% *p* < 0.0001). When taking all detected amplifications into consideration, the average amplification calling per sample was nearly 5-fold lower in the 10–30% group compared to the 61–95% group (11.7% vs. 52.4%; *p* < 0.0001).

### 3.4. Tumors with High MAPD Score

Considering that the MAPD score is one of the most crucial QC metrics in determining the calling of CNV, we set our cut-off at 0.43 (mean ± 2SD) to ensure that 5% of outliers with high MAPD scores will be flagged during data analysis using OCAv3. Notably, our MAPD score cut-off (0.43) is slightly lower than the 0.5 cut-off score suggested by the vendor (Table 1). Out of 756 tumors analyzed, 2 samples (one colon and one lung) had MAPD scores of 0.46 and 0.44, respectively. NGS detected six copies of *ERBB2* in each of these samples. According to our in-house testing protocol, tumors showing *ERBB2* amplification but with a MAPD score above 0.43 require confirmation by IHC and/or FISH. Subsequently, both tumors were confirmed to lack *ERBB2* amplification by FISH, with *ERBB2* to *CEP17* ratios of 1 and 1.8, respectively. These cases were identified as false positive NGS results in the setting of higher MAPD scores.

### 3.5. The Analytical Performance of OCAv3 in Detecting ERBB2 Amplification in Uterine Carcinoma

The analytical performance of the OCAv3 NGS assay for detecting *ERBB2* amplifications was benchmarked against the gold standard HER2 testing method, IHC-FISH, across two independent cohorts of uterine carcinomas. The first cohort, serving as the discovery set, included 121 uterine carcinoma cases tested for HER2 between February 2021 and March 2024. The second cohort, used for validation, comprised 29 cases tested for HER2 between April 2024 and September 2024.

In the discovery cohort, the overall HER2-positivity rate was 28.1% (34/121). HER2-negative cases accounted for 28.9% (35/121) IHC 0, 16.5% (20/121) IHC 1+, and 26.4% (32/121) IHC 2+ with negative FISH results. HER2-positive cases included 12.4% (15/121) IHC 2+ with FISH amplification and 15.7% (19/121) IHC 3+. Notably, 82.6% of the cases in the discovery cohort were p53-abnormal, with 0.8% equivocal, 13.2% wild-type and 3.3% cases of unknown p53 status. Among these, 6.6% were FIGO grade 1 or 2 endometrioid adenocarcinomas, while 91.7% were high-grade endometrial cancers, including FIGO grade 3 endometrioid carcinomas, serous carcinomas, mixed carcinomas with a serous component, high grade endometrial carcinomas with ambiguous features, carcinosarcomas, gastric-type carcinomas of the endometrium, undifferentiated/dedifferentiated carcinomas and mesonephric-like endometrial adenocarcinomas, and 1.7% of cases had unknown histology.

**Figure 2:**
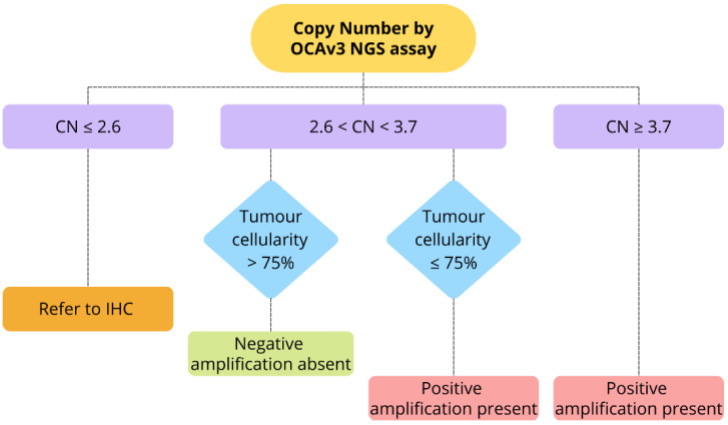
Decision algorithm for *ERBB2* amplification classification based on the Oncomine Comprehensive Assay v3 (OCAv3) copy number (CN) and tumor cellularity in uterine carcinoma. We propose a future HER2/*ERBB2* testing algorithm in which IHC remains the first-tier test, while NGS is used for molecular classification and detection of *ERBB2* amplifications. Cases with NGS-detected amplification will be classified as positive. For NGS-negative cases with equivocal IHC and/or low tumor cellularity, confirmatory FISH testing will be recommended. The 2.6–3.7 CN range may be considered a “gray zone” requiring further FISH evaluation.

*ERBB2* amplifications were initially defined by a threshold of CN > 4 (as outlined in Methods Section 2.7). In the discovery cohort, this threshold yielded a sensitivity of 26%, specificity of 100%, positive predictive value (PPV) of 100%, and negative predictive value (NPV) of 78%, compared to HER2 IHC-FISH. To improve sensitivity, while maintaining 100% specificity, the CN threshold and tumor cellularity cutoffs were manually refined. With the refined threshold, samples with a CN ≤ 2.6 were classified as negative regardless of tumor cellularity (Figure 2). For CN values between >2.6 and <3.7, samples were considered positive if tumor cellularity was ≤75% and negative if >75%. Samples with CN ≥ 3.7 were deemed positive irrespective of cellularity. Using this refined threshold, sensitivity in the discovery cohort increased to 79% and NPV to 93%, while specificity and PPV remained at 100% (*p* < 0.0001, Table 7).

The new refined threshold was then validated in a second cohort, which had an overall HER2-positivity rate of 27.6% (8/29). HER2-negative cases included 31.0% (9/29) IHC 0, 27.6% (8/29) IHC 1+ and 13.8% (4/29) IHC 2+ with negative FISH results. HER2-positive cases included 6.9% (2/29) IHC 2+ with FISH amplification and 20.7% (6/29) IHC 3+. This cohort included 75.9% p53-abnormal, 20.7% wild-type and 3.4% of cases with unknown p53 status. Histologically, 3.4% were FIGO grade 2 endometrioid adenocarcinomas, while 96.6% were high-grade endometrial cancers, including FIGO grade 3 endometrioid carcinomas, serous carcinomas, carcinosarcomas and dedifferentiated carcinomas. In the validation cohort, the refined threshold demonstrated a sensitivity of 88%, an NPV of 95%, and maintained a specificity and PPV of 100% (*p* < 0.0001), similar to the results in the discovery cohort.

Next, we focused on the analysis of HER2 IHC 2+ cases, as they necessitate FISH testing, an approach that is both time- and resource-intensive. In the discovery cohort, there were 47 HER2 IHC 2+ cases, with 32 non-amplified and 15 amplified by FISH (Table 7), while the validation cohort included 6 HER2 IHC 2+ cases, with 4 non-amplified and 2 amplified. In the discovery cohort, 26 of 31 cases with CN ≤ 2.6 and *ERBB2*/CEP17 ratio < 2.0 were true negatives, while five were false negatives. For 16 cases with CN > 2.6, the OCAv3 assay accurately classified all: six cases with CN > 2.6 to < 3.7 and tumor cellularity > 75% were negative, six cases with CN > 2.6 to <3.7 and cellularity ≤ 75% were positive, and four cases with CN ≥ 3.7 were positive (Table 7). In the validation cohort, applying the cut-off established in the discovery cohort on six HER2 IHC 2+ cases (see Figure 1), four of five cases with CN ≤ 2.6 were accurately classified as true negatives, while one was a false negative. The case with CN > 2.6 was correctly identified as a true positive. Thus, this adjusted threshold could potentially eliminate the need for FISH in 16/47 of equivocal HER2 IHC 2+ uterine carcinomas in the discovery cohort and 1/6 in the validation cohort, for a total of 17/53 (32.1%) cases. However, due to the limited sample size, particularly in the 2.6–3.7 CN range, further investigation is warranted. This CN range may be defined as a “gray zone” requiring confirmatory FISH testing.

## 4. Discussion

In this study, we comprehensively assessed the performance of two targeted amplicon-based NGS panels (OFA and OCAv3) in detecting gene amplifications across various tumor types to facilitate personalized cancer treatments [17]. Additionally, we provided real-world data on the application of these panels in assessing *ERBB2* amplification in different tumor types, including uterine carcinoma. Our findings indicate that when standardized QC metrics, such as the MAPD score [18], are met, small amplicon-based targeted NGS assays, such as OFA and OCAv3, can be applied to FFPE samples to detect gene amplification with a near-perfect PPV. Their major limitation, however, is the reduced sensitivity in detecting gene amplifications in samples with low tumor cellularity or in tumors with amplifications ranging from four to seven copies. Our results indicate a nearly five-fold lower detection rate when tumor cellularity is below 30%, suggesting a high likelihood of missing amplifications in these samples. Of note, SNP microarray CN estimates are also influenced by tumor cellularity in the same manner as NGS CN estimates. It is, therefore, crucial when reporting CN results, that the limitations of sensitivity be clearly stated in the final report.

Building on these findings, our proof-of-concept study also demonstrates the broader applicability of amplicon-based NGS for gene amplification detection in clinical settings, particularly in the context of targeted therapies. By offering practical insights for laboratories utilizing the OCAv3 or OFA assays, we provide a valuable validation framework that can support the integration of this approach into routine molecular testing. Laboratories adhering to the minimum QC cutoffs outlined in Table 1 may find the thresholds described here transferable and applicable to their own settings. Furthermore, labs can adapt and tweak the framework we presented to fit their specific requirements, ensuring optimized NGS assays for accurate gene amplification detection.

This study highlights key technical considerations [19,20,21,22,23,24], particularly the role of MAPD in ensuring accurate gene amplification detection. The MAPD metric, widely used by SNP microarrays to assess the quality of CNV calls, measures read coverage noise across all amplicons in a panel [15]. Higher signal noise across multiple amplicons increases the likelihood of misinterpretation of CNV calls. As such, we have implemented a reflex testing protocol wherein *ERBB2* amplification detected in samples with a MAPD score above 0.43 should be confirmed by IHC and/or FISH (the current gold standard method [25]). In our first cohort (34 FFPE samples), we purposefully included 2 tumor samples (samples 32 and 33 in Table 3) with *ERBB2* amplification but high MAPD scores (0.45 and 0.46, respectively) for orthogonal testing. Both samples were subsequently confirmed positive for *ERBB2* amplifications by FISH. By contrast, two other samples from our cohort of 756 tumors were found to be negative for *ERBB2* amplification by FISH. This underscores the importance of individual laboratories adjusting and validating MAPD score cut-off to minimize the likelihood of reporting false positive results.

In addition to the MAPD score, our findings underscore the significance of considering tumor cellularity for accurate gene amplification detection using amplicon-based NGS assays. Our results indicate that by taking tumor cellularity into account, the CN cut-off can be adjusted to below four, thereby maximizing the performance of NGS assays for detecting *ERBB2* gene amplification. In current clinical practice, tumor cellularity is not typically integrated into amplicon-based NGS bioinformatics pipelines. Nonetheless, we recommend that laboratories consider this factor when interpreting results. We recognize that tumor cellularity enumeration is currently subject to considerable variability depending on the pathologist’s assessment and sample quality [26]. However, in the era of digital pathology, there is potential for more precise and standardized assessments of tumor cellularity using artificial intelligence-based deep neural networks, as these technologies continue to evolve.

Additional consideration for the clinical implementation of amplicon-based NGS assays is the preanalytical variability between different sample types, such as biopsies and excisions, and how these factors affect gene amplification detection. Excisions involve larger samples, but may introduce variability due to uncontrolled preanalytical factors, whereas biopsies are smaller, but offer better preanalytical conditions. Further, addressing intratumoral heterogeneity in endometrial tumors is crucial, particularly whether amplicon-based NGS assays can detect *ERBB2* amplification if only a small portion of the tumor is amplified, even with overall tumor cellularity greater than 30%. This has been discussed in detail in our recent publication [5]. Therefore, preanalytical factors, tumor cellularity, CN and intratumoral heterogeneity need to be all considered collectively to ensure the accurate application of NGS in real-world clinical settings [27]. Finally, we recognize that there are broader preanalytical, analytical and post-analytical challenges in implementing clinical NGS testing in general. Detailed discussion would fall beyond the scope of our study. For further insights, readers could refer to an excellent review article [28].

Given the increasing implementation of NGS-based molecular profiling in advanced and metastatic solid tumors, there has been growing interest in using amplicon-based NGS to concurrently screen for gene amplifications [3,5,6,12,13]. In this study, we focused on uterine carcinomas, where HER2 testing by IHC/FISH is standard practice. Looking forward, we envision a future where a new algorithm for HER2/*ERBB2* testing in uterine tumors is implemented, with IHC and molecular results becoming available within 7–10 days. NGS testing for the molecular classification of uterine carcinomas, including *POLE* tumor profiling, is currently routinely performed and funded by the public healthcare system in numerous jurisdictions, including Canada. The OCAv3 NGS assay offers a more time-, cost- and tissue-efficient method for simultaneously assessing *POLE* mutations and *ERBB2* amplifications in uterine carcinomas, particularly in cases with limited tissue material. In this new *ERBB2* testing algorithm, cases with NGS-detected amplification will be categorized as positive. If NGS results are negative based on the adjusted cut-off and IHC is equivocal, FISH testing will follow if tumor cellularity is low. Our findings suggest that NGS could reduce the need for FISH testing in 16/47 of HER2 IHC 2+ tumors in the discovery cohort and 1/6 in the validation cohort, for a total of 17/53 (32.1%) cases. However, given the limited sample size in this study, especially within the 2.6–3.7 CN range, further investigation is needed. This CN range may be defined as a “gray zone” requiring confirmatory FISH testing. This approach could save time and costs for diagnostic laboratories, streamlining workflows and enabling timely targeted therapy decisions. Additionally, using a validated NGS testing algorithm could allow molecular classification and identification of patients for HER2-targeted therapies in cancers not routinely tested by HER2 IHC. This includes cancers with low HER2 positivity rates or those not typically considered for anti-HER2 treatments, expanding therapeutic options and potentially positively impacting patient care.

A limitation of this study is the small cohort size. Larger validation studies involving more IHC 2+ tumors are recommended to confirm the applicability of this study’s findings. It remains to be determined whether the cutoff described here can be extended to other tumor types, as intratumor heterogeneity in HER2 expression and amplification can vary significantly across different solid tumors.

## 5. Conclusions

In summary, our study demonstrates the major strengths and limitations of amplicon-based NGS assays in detecting gene amplifications. By adjusting the threshold of calling amplifications and taking tumor cellularity into consideration, it is possible to increase the sensitivity in detecting *ERBB2* amplification to about 80%, while maintaining 100% specificity. To reduce the likelihood of false positive CN calling when using amplicon-based NGS assays, we recommend that the MAPD score should be routinely assessed, and tumor cellularity should be included in the assessment.

## Figures and Tables

**Table 1:** Quality control metrics for Oncomine next-generation sequencing panels.

**NGS Panel**	**Mapped Reads**	**On Target (%)**	**Mean Depth**	**Uniformity (%)**	**Mean Read Length**	**MAPD** **(Mean ± 2SD)**
OFA	506,869	98.28	1824	99.11	116	0.38 ± 0.084 *
OCAv3	6,214,804	95.10	1788	95.72	110	0.30 ± 0.133 *
**Recommended Quality Control Metrics**						
OFA	≥300,000	N/A	≥675	≥80%	≥75 bp	<0.5
OCAv3	≥3,000,000	N/A	≥800	≥80%	≥75 bp	<0.5

* The cut-off scores for median MAPD were established at 0.46 for OFA and 0.43 for OCAv3 for our in-house testing protocol. These thresholds were designed to flag outliers with elevated MAPD scores during data analysis. NGS, next-generation sequencing; MAPD, median absolute pairwise difference; SD, standard deviation.

**Table 4:** Limits of detection of gene amplification by the Oncomine next-generation sequencing panels Oncomine Focus Assay and Oncomine Comprehensive Panel.

	Reference	Expected CN	Average MAPD	*FGFR3* CN	*MYC* CN	*ERBB2* CN	*MYCN* CN	*EGFR* CN	*MET* CN
**OFA**	NEAT (100%)	14	0.31	12.33	9.73	13.95	17.36	16.71	18.31
	50% Dilution	8	0.33	6.12	5.65	6.64	8.34	8.52	10.78
	25% Dilution	5	0.36	4.22	5.06	4.66	4.95	5.16	6.55
	12.5% Dilution	3.5	0.37	3.15	3.51	3.23	3.54	3.65	4.5
**OCAv3**	NEAT (100%)	14	0.22	13.63	9.62	15.72	14.64	14.8	14.26
	50% Dilution	8	0.22	5.91	5.37	7.06	6.93	7.5	8.11
	25% Dilution	5	0.24	4.62	3.21	4.03	4.61	5.11	4.14
	12.5% Dilution	3.5	0.24	2.35	3.61	3.24	2.88	3.34	3.83

MAPD, median absolute pairwise difference; OCAv3, Oncomine Comprehensive Panel; OFA, Oncomine Focus Assay; CN, copy number.

**Table 5:** Frequency of detection of gene amplifications across different tumor types.

Tumor Type	Cases, N	Cases with Amplification, N (%)	# of Amplifications Detected
Breast cancer	13	5 (38.5)	22
Colorectal cancer	242	42 (17.4)	73
Endometrial cancer	135	42 (31.1)	72
Lung cancer	142	34 (23.9)	50
Melanoma	153	18 (11.8)	37
Prostate cancer	63	9 (14.3)	15
Total	748	150 (20.0)	269

The cohort consists of 756 cases; however, only 748 cases are listed in Table 4, as the remaining 8 cases represent rare occurrences of specific cancers (biliary, bladder, glioma, and thyroid), with one to four cases of each.

**Table 6:** Detection rate of gene amplifications stratified by tumor cellularity.

Tumor Cellularity	Total Cases	Amplification Cases, N (%)	All Amplification Calls, N (%)	*p*-Value
10–30%	163	15 (9.2)	19 (11.7)	<0.0001
31–60%	301	62 (20.6)	112 (37.2)	
61–95%	292	78 (26.7)	153 (52.4)	

**Table 7:** Comparison of HER2 IHC-FISH status and *ERBB2* copy number results from the Oncomine Comprehensive Panel in uterine carcinomas using refined thresholds.

IHC	FISH	IHC-FISH Status	Copy Number on OCAv3							
HER2	*ERBB2/CEP17*		≤2.6		>2.6 to <3.7				≥3.7	
					Tumor Cellularity		Tumor Cellularity			
					>75%,		≤75%,			
			OCAv3 Result: Negative				OCAv3 Result: Positive			
			Discovery N (%)	Validation N (%)	Discovery N (%)	Validation N (%)	Discovery N (%)	Validation N (%)	Discovery N (%)	Validation N (%)
0–1+	N/A	Negative	54 (44.6)	17 (58.6)	1 (0.8)	0	0	0	0	0
2+	<2		26 (21.5)	4 (13.8)	6 (5.0)	0	0	0	0	0
2+	≥2.0	Positive	5 (4.1)	1 (3.4)	0	0	6 (5.0)	1 (3.4)	4 (3.3)	0
3+	N/A		2 (1.7)	0	0	0	3 (2.5)	0	14 (11.6)	6 (20.7)

FISH, fluorescence in situ hybridization; IHC, immunohistochemistry; OCAv3, Oncomine Comprehensive Panel. The ‘+’ reflects how the IHC is scored (e.g., ‘1+’, ‘2+’).

## Data Availability

The data that support the findings of this study are available on request from the corresponding author, W.-Y.H.
